# Supplementary material for: HSPCs display within-family homogeneity in differentiation and proliferation despite population heterogeneity
Source: eLife. 2021 May 18;10:e60624. doi: 10.7554/eLife.60624 (PMC8175087; doi:10.7554/eLife.60624)
Supplement: Source code 1. [file elife-60624-code1.zip › Generational_multiplex_analysis-master/R_script_for_Jonckheere_test/Note_on_csv_files.docx]

Csv files for Jonckheere_test.R were created pooling information from the index sorting files and the single cell analysis after culture.

Each row represents a family that has been observed in the “Single cell” data.

Maxdd column indicated the maximum generation reached by that family after culture.

Marker columns ckit, SCA-1, Flt3, SLAM and CD48 store the expressions at sort from the seeded progenitor.

Columns SLAM-HSC, ST-HSC, MPP, SLAM+ Flt3+, GMP, MEP, SLAM+ MEP, MP and CD16/32- ckit- store a binary variable that is 1 if the family, after culture, was found with at least one of the cell type relative to the column, 0 otherwise.
