# Supplementary material for: HSPCs display within-family homogeneity in differentiation and proliferation despite population heterogeneity
Source: eLife. 2021 May 18;10:e60624. doi: 10.7554/eLife.60624 (PMC8175087; doi:10.7554/eLife.60624)
Supplement: Transparent reporting form [file elife-60624-transrepform.pdf]

eLife's transparent reporting form

We encourage authors to provide detailed information *within their submission* to facilitate the interpretation and replication of experiments. Authors can upload supporting documentation to indicate the use of appropriate reporting guidelines for health-related research (see EQUATOR Network), life science research (see the BioSharing Information Resource), or the ARRIVE guidelines for reporting work involving animal research. Where applicable, authors should refer to any relevant reporting standards documents in this form.

If you have any questions, please consult our Journal Policies and/or contact us: editorial@elifesciences.org.

Sample-size estimation

- You should state whether an appropriate sample size was computed when the study was being designed
- You should state the statistical method of sample size computation and any required assumptions
- If no explicit power analysis was used, you should describe how you decided what sample (replicate) size (number) to use

Please outline where this information can be found within the submission (e.g., sections or figure legends), or explain why this information doesn't apply to your submission:

We did not compute a sample-size estimation. The required number of experiments was based on previous experiences with a similar experimental setup (e.g. Horton et al. 2018).

Replicates

- You should report how often each experiment was performed
- You should include a definition of biological versus technical replication
- The data obtained should be provided and sufficient information should be provided to indicate the number of independent biological and/or technical replicates
- If you encountered any outliers, you should describe how these were handled
- Criteria for exclusion/inclusion of data should be clearly stated
- High-throughput sequence data should be uploaded before submission, with a private link for reviewers provided (these are available from both GEO and ArrayExpress)

Please outline where this information can be found within the submission (e.g., sections or figure legends), or explain why this information doesn't apply to your submission:

The number of times experiments were performed can be found in the methods section of the manuscript.
The total number of cells analyzed can be found in the results section.
The data itself can be found as a supplemental table, the number of replicates for each experiment can be found in supplemental table 2.
Outliers were not excluded from the analysis.
There were no inclusion/exclusion criteria for our manuscript, except for the exclusion of cell families with an impossible number of members as described in the results section.

Statistical reporting

- Statistical analysis methods should be described and justified
- Raw data should be presented in figures whenever informative to do so (typically when N per group is less than 10)
- For each experiment, you should identify the statistical tests used, exact values of N, definitions of center, methods of multiple test correction, and dispersion and precision measures (e.g., mean, median, SD, SEM, confidence intervals; and, for the major substantive results, a measure of effect size (e.g., Pearson's r, Cohen's d)
- Report exact p-values wherever possible alongside the summary statistics and 95% confidence intervals. These should be reported for all key questions and not only when the p-value is less than 0.05.

Please outline where this information can be found within the submission (e.g., sections or figure legends), or explain why this information doesn't apply to your submission:

The statistical analysis methods for each figure is described in in the results section of the manuscript, in the figure legend as well as in the legend of the accompanying supplementary table. Detailed information on the statistical analysis method are provided in the methods and supplementary methods sections.
Due to the large number of cell analysed, plotting all datapoints was not possible in most figures. However, figure 2A depicts the family membership, cell phenotype and number of divisions each cell has undergone.
The exact N and p-values for each statistical test can be found in supplementary tables 2-7. A description of the multiple test correction used (Holm-Bonferroni) is also provided in the supplementary methods.
Definitions for each statistic are given in the main text, figure legends and in the legend of the supplementary tables accompanying each figure.

(For large datasets, or papers with a very large number of statistical tests, you may upload a single table file with tests, Ns, etc., with reference to sections in the manuscript.)

Group allocation

- Indicate how samples were allocated into experimental groups (in the case of clinical studies, please specify allocation to treatment method); if randomization was used, please also state if restricted randomization was applied
- Indicate if masking was used during group allocation, data collection and/or data analysis

Please outline where this information can be found within the submission (e.g., sections or figure legends), or explain why this information doesn't apply to your submission:

This does not apply to our submission as we did not allocate samples/individuals to experimental groups.
Cells were allocated to experimental conditions at random. Data collection and analysis were all automated and, therefore, were not masked.

Additional data files ("source data")

- We encourage you to upload relevant additional data files, such as numerical data that are represented as a graph in a figure, or as a summary table
- Where provided, these should be in the most useful format, and they can be uploaded as "Source data" files linked to a main figure or table
- Include model definition files including the full list of parameters used

- Include code used for data analysis (e.g., R, MatLab)
- Avoid stating that data files are "available upon request"

Please indicate the figures or tables for which source data files have been provided:

The source data are included in the supplemental file named "source data", including an Excel file with all the data used for figure 2-3 and the code used to analyze the data For the latest version of the code used please check https://github.com/GiulioPr/Generational_multiplex_analysis.
A model to simulate different scenarios of clonal concordance, previously defined in (Marchingo et al. 2016), is described in the supplementary methods (section Beta-binomial model for clonal concordance), together with its parameters.
